# What do patients and dermatologists prefer regarding low-risk basal cell carcinoma follow-up care? A discrete choice experiment

**DOI:** 10.1371/journal.pone.0249298

**Published:** 2021-03-29

**Authors:** Sven van Egmond, Esther de Vries, Loes Hollestein, Maarten Bastiaens, Kees-Peter de Roos, Daniëlle Kuijpers, Ewout Steyerberg, Marlies Wakkee, Klara Mosterd, Tamar Nijsten, Esther W. de Bekker-Grob

**Affiliations:** 1 Department of Dermatology, Erasmus MC Cancer Institute, Rotterdam, The Netherlands; 2 Department of Clinical Epidemiology and Biostatistics, Pontificia Universidad Javeriana, Hospital San Ignacio, Bogota, Colombia; 3 Department of Public Health, Erasmus MC University Medical Center, Rotterdam, The Netherlands; 4 Department of Dermatology, TweeSteden Ziekenhuis, Tilburg, The Netherlands; 5 Department of Dermatology, DermaPark, Uden, The Netherlands; 6 Department of Dermatology, Amphia Ziekenhuis, Breda, The Netherlands; 7 Department of Biomedical Data Sciences, Leiden University Medical Center, Leiden, The Netherlands; 8 Department of Dermatology, Maastricht University Medical Centre, Maastricht, The Netherlands; 9 Section of Health Technology Assessment & Erasmus Choice Modelling Centre, Erasmus School of Health Policy & Management, Erasmus University, Rotterdam, The Netherlands; University of Queensland Diamantina Institute, AUSTRALIA

## Abstract

**Background:**

Follow-up after low-risk basal cell carcinoma (BCC) is being provided more frequently than recommended by guidelines. To design an acceptable strategy to successfully reduce this ‘low-value’ care, it is important to obtain insights into the preferences of patients and dermatologists.

**Objective:**

To determine the preferences and needs of patients and dermatologists to reduce low-risk BCC follow-up care, and the trade-offs they are willing to make.

**Methods:**

A questionnaire including a discrete choice experiment was created, containing attributes regarding amount of follow-up, continuity of care, method of providing addition information, type of healthcare provider, duration of follow-up visits and skin examination. In total, 371 BCC patients and all Dutch dermatologists and dermatology residents (n = 620) were invited to complete the questionnaire. A panel latent class model was used for analysis.

**Results:**

Eighty-four dermatologists and 266 BCC patients (21% and 72% response rates respectively) completed the discrete choice experiment. If the post-treatment visit was performed by the same person as treatment provider and a hand-out was provided to patients containing personalised information, the acceptance of having no additional follow-up visits (i.e. following the guidelines) would increase from 55% to 77% by patients. Female patients and older dermatologists, however, are less willing to accept the guidelines and prefer additional follow-up visits.

**Limitations:**

The low response rate of dermatologists.

**Conclusion:**

This discrete choice experiment revealed a feasible strategy to substantially reduce costs, while maintaining quality of care, based on the preferences and needs of BCC patients, which is supported by dermatologists.

## Introduction

Basal cell carcinoma (BCC) is a subtype of keratinocyte cancer and the most common type of cancer among Caucasians [1]. The probability that a BCC metastasizes or causes death is low, but BCCs can cause significant morbidity by local spread. Most BCCs are treated by a relatively simple excision or even topical treatment for certain low-risk BCCs [2, 3].

The high and rising incidence of BCC puts healthcare systems under pressure [4]. To manage finite resources, low-value care (i.e. care which provides no or little clinical benefit to patients) should be reduced [5]. Current European and Dutch guidelines recommend to only provide annual follow-up care to high-risk BCC patients (i.e. high risk for recurrence or already treated for recurrent BCC and patients with history of multiple BCCs), because there is no evidence that increased follow-up care leads to improved health outcomes [6, 7]. The guidelines state that it is reasonable to provide one post-treatment visit for low-risk BCC patients to counsel them about sun protection measures and to stress the importance of self-monitoring for possible local recurrence and new skin cancers. Any follow-up visits after the post-treatment visit after low-risk BCC can therefore be considered as low-value care. However, a recent study shows that Dutch dermatologists still provide this “low-value follow-up care” to 83% of low-risk BCC patients [8].

Qualitative interviews with dermatologists and focus groups with BCC patients revealed the factors influencing this low-value care, such as the wishes of patients to remain in dermatological follow-up (because they experts specialised in skin care, as opposed to GPs, who are generalists) and dermatologists complying with patients wishes [9, 10]. In order to design a successful strategy to de-adopt low-value BCC follow-up care, it is important to learn how important each of these factors are to the patients and dermatologists. Therefore, this study aimed to quantify the preferences of BCC patients and dermatologists, and to determine which trade-offs they are willing to make to accept a reduction of low-risk BCC follow-up.

## Methods

### Study sample

#### BCC patients

Dutch speaking patients, 18 years or older, presenting at the dermatologists with a lesion that was clinically suspicious of BCC or recent biopsy confirmed BCC were approached by their treating dermatologist to participate. These were patients from one university hospital (Erasmus MC), three general hospitals (Elisabeth-TweeSteden Hospital, Amphia Hospital, Bravis Hospital) and two independent sector treatment centres (DermaPark, Mohs Klinieken) in the Netherlands. Patients received an envelope containing information about the study, an informed consent form, a questionnaire which included a Discrete Choice Experiment (DCE) (S1 Appendix) and a pre-paid return envelope. All patients who agreed to participate returned a signed informed consent form to participate in the current study, which was approved by the Medical Ethical Committee of the Erasmus MC (MEC-2014-374). Patients were excluded afterwards if the pathologist concluded that the lesion was not a BCC.

#### Dermatologists

All dermatologists and dermatology residents in the Netherlands (from here onward both mentioned as dermatologists) received an email with a digital link to the questionnaire containing the same DCE as the patients (S2 Appendix). Additionally, dermatologists were encouraged to complete the questionnaire during a national conference.

### DCE: Attributes and levels

The DCE concept originates from mathematical psychology, but has increasingly found its way into healthcare [11]. A DCE repeatedly presents varying alternative situations to respondents and asks them to choose their most preferred alternative [12]. The DCE method assumes that each alternative can be described by their characteristics (“attributes”), that the respondent’s valuation of the alternative depends on the levels of these attributes, and that the choices are based on a latent utility function [13]. In a DCE, preferences for health outcomes and non-health outcomes can be taken into account simultaneously [14]. This makes the DCE an ideal method to measure preferences for aspects of low-risk BCC follow-up visits, and to determine how to reduce this care without trial-and-error-implementation.

In addition to a literature review,[15–21] we constructed our DCE based on focus groups with BCC patients and interviews with dermatologists to carefully detect and select relevant attributes [9, 10, 13]. This ultimately led to the final determination of six attributes (Table 1).

**Table 1:** Attributes and levels of the discrete choice experiment on low-risk BCC follow-up care.

Attributes	Attribute Levels
**Standard post-treatment visit performed:**	Not by same person as treatment provider (Ref)
	By the same person as treatment provider
**In addition to oral information, extra information^1^ will be provided by:**	E-health (Ref)
	Personalised Letter
	General hand-out
	General website
**The additional follow-up visit(s) will be planned:**	1 year after treatment (Ref)
	6 and 12 months after treatment^2^
	1 and 2 years after treatment
**The additional follow-up visit(s) will be conducted by:**	Nurse practitioner (Ref)
	General practitioner
	Dermatologist
**The duration of the additional follow-up visit(s) will be:**	5 minutes (Ref)
	10 minutes
	15 minutes
**Part of skin to be checked during the additional follow-up visits**	Face, upper body and treated area (Ref)
	Full body

Abbreviations: Ref, Attribute of reference.

^1^Extra information about severity of disease, prognosis, further treatment and/or follow-up and self-examination instructions. Which could be general information, or personalised via E-health or personalised letter.

^2^Both visits within the time frame of one diagnosis-related group (DRG).

### Study design and questionnaire

**Fig 1:**
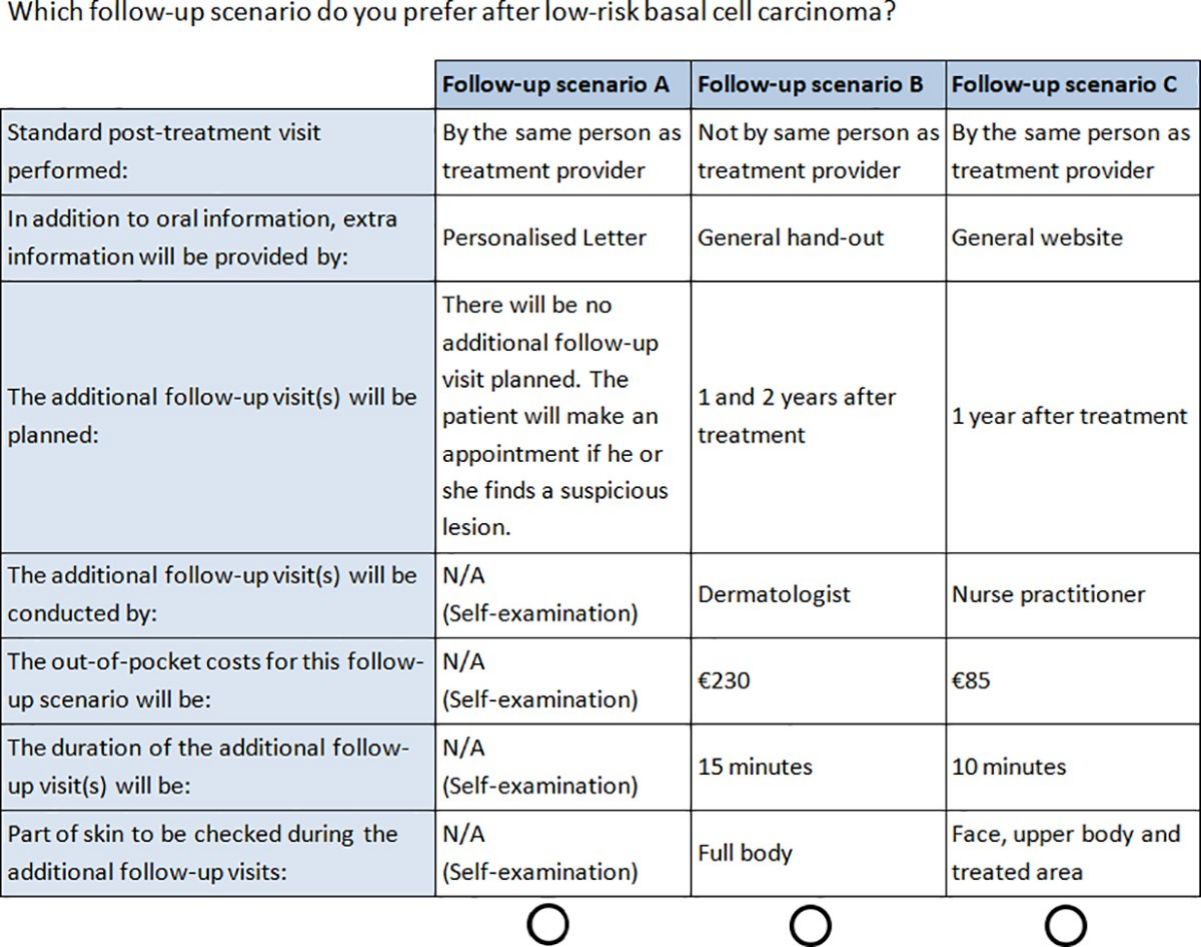
Choice task example of the discrete choice experiment.

Based on the six attributes and their levels, many different choice alternatives and choice tasks can be generated. It is not feasible to present all possible combinations to a respondent without creating an unreasonably long questionnaire. In order to achieve maximum efficiency from the minimum amount of ‘choice tasks’ needed (i.e. to counterbalance statistical needs and the burden for the respondent), a D-efficient design was created using Ngene software (http://www.choice-metrics.com) [22]. We took two-way interactions between the attributes ‘The additional follow-up visit(s) will be planned’ and ‘The additional follow-up visit(s) will be conducted by’ into account, based on the outcomes of the qualitative study. We created a set of 24 choice tasks (Fig 1), which was blocked [14] in two sets of twelve for BCC patients and a full set was presented to dermatologists, as we expected the sample size as well as the response rate of dermatologists to be smaller. Each choice task contained three alternatives: two alternatives containing BCC follow-up schemes and one alternative to choose ‘no additional BCC follow-up’ (i.e. following the guideline).

In addition, to simulate real life choices as close as possible, the corresponding real-life costs of each follow-up scenario were shown. Each attribute was explained in the survey before the choice tasks were presented. To test choice consistency, an extra choice task 13 was included, which was identical to choice task 4. Demographic questions were added to the questionnaire. The questionnaire including the DCE was pilot tested using think-aloud technique on a random group of 22 BCC patients and ten dermatologists, where they could ask for clarification when needed and to test if the length was acceptable. The DCE was clear to these patients and dermatologists and did not lead to any changes.

### Sample size

The results of the pilot were used to determine that 250 BCC patients were required to complete the DCE [23]. By taking an expected response rate of 70% into account [24–26], we invited 371 patients. As the response rate of physicians is notoriously low [27–29], we invited all dermatologists and dermatology residents (n = 620) in the Netherlands.

### Statistical analyses

The analyses were conducted by using Stata 4.2 (http://www.stata.com) and NLOGIT 5.0 (Econometric Software Inc [30]). Each choice task was considered as one observation. To take the sample size, the fact that one respondent completed 12–24 choice tasks, the model fit and our interest in preference heterogeneity into account, a panel latent class analysis (LCA) was conducted for the final analyses. An LCA determines whether patterns in preferences (i.e. latent classes of preferences) exist and provides a modelled probability for each respondent to belong in a certain class [31]. Demographic covariates retrieved from the questionnaire were linked to the probability that respondents belonged to a specific class, in order to determine the composition of the classes. Taking the smaller sample size of dermatologists into account, without demographic covariates that could be linked to specific classes, a more simplistic model (Multinomial logit with interaction terms of the demographic covariates with the attribute-levels) was conducted. We performed a sensitivity test by excluding respondents who failed the consistency test (i.e. choosing different alternatives on the identical choice tasks) from the analyses. After testing for two-way interactions and attribute linearity, the optimal latent class utility function was:

Scenario with additional follow-up visits:

$${V(alt1)}_{nsj|c}=\beta_{1|c}\;{same\;healthcare\;professional}_{nsj|c}+\beta_{2|c}\;{additional\;information\;by\;personalised\;letter}_{nsj|c}+\beta_{3|c}\;{additional\;information\;by\;general\;handout\;}_{nsj|c}+\beta_{4|c}{{\;additional\;information\;by\;general\;website}_{nsj|c}+\beta_{5|c}\;additional\;followup\;at\;6\;and\;12\;months\;after\;treatment}_{nsj|c}+\beta_{6|c}{\;additional\;followup\;at\;1\;and\;2\;years\;after\;treatment}_{nsj|c}+\beta_{7|c}\;{additional\;followup\;conducted\;by\;general\;practitioner\;}_{nsj|c}+\beta_{8|c}\;{additional\;followup\;conducted\;by\;dermatologist}_{nsj|c}+\beta_{9|c}\;{duration\;of\;additional\;followup\;visit\;10\;minutes}_{nsj|c\;}+\beta_{10|c}\;{duration\;of\;additional\;followup\;visit\;15\;minutes\;}_{nsj|c}+\beta_{11|c}\;{inspected\;skin\;part\;full\;body\;}_{nsj|c}$$

$${V(alt2)}_{nsj|c}=\beta_{1|c}\;{same\;healthcare\;professional}_{nsj|c}+\beta_{2|c}\;{additional\;information\;by\;personalised\;letter}_{nsj|c}+\beta_{3|c}\;{additional\;information\;by\;general\;handout}_{nsj|c}+\beta_{4|c}{{\;additional\;information\;by\;general\;website}_{nsj|c}+\beta_{5|c}\;additional\;followup\;at\;6\;and\;12\;months\;after\;treatment}_{nsj|c}+\beta_{6|c}{\;additional\;followup\;at\;1\;and\;2\;years\;after\;treatment}_{nsj|c}+\beta_{7|c}\;{additional\;followup\;conducted\;by\;general\;practitioner\;}_{nsj|c}+\beta_{8|c}\;{additional\;followup\;conducted\;by\;dermatologist}_{nsj|c}+\beta_{9|c}\;{duration\;of\;additional\;followup\;visit\;10\;minutes}_{nsj|c\;}+\beta_{10|c}\;{duration\;of\;additional\;followup\;visit\;15\;minutes\;}_{nsj|c}+\beta_{11|c}\;{inspected\;skin\;part\;full\;body\;}_{nsj|c}$$

Scenario without additional follow-up visits:

$${V(alt3)}_{nsj|c}=\beta_{12|c}+\beta_{13|c}{same\;healthcare\;professional\;}_{nsj|c\;}+\beta_{14|c}\;{additional\;information\;by\;personalised\;letter}_{nsj|c}+\beta_{15|c}\;{additional\;information\;by\;general\;handout}_{nsj|c}+\beta_{16|c}{additional\;information\;by\;general\;website}_{nsj|c}$$

Where

**Table:** 

V_nsj|c_	represents the observable utility that respondent ‘n’ belonging to class segment ‘c’ has for alternative ‘j’ in choice set ‘s’;
*alt*	represent the three alternatives in the choice set;
*β*_12|c_	represent an alternative-specific constant for the ‘no additional BCC follow-up scenario’ compared to the ‘additional BCC follow scenario’ for a certain class;
*β*_1–11|c_	are class-specific parameter weights (coefficients) associated with the attributes of the DCE to provide additional follow-up;
*β*_13–16|c_	are class-specific parameter weights (coefficients) associated with the attributes of the DCE to not provide additional follow-up.

A statistically significant coefficient of an attribute(level) shows that the attribute is important for the respondents in their decision to accept a certain BCC follow-up. The sign of the coefficient shows whether they prefer (positive sign) or disfavour (negative sign) the particular level of the attribute. The size of the coefficients indicates the relative importance (bigger equals more important).

### Expected choice probability of BCC follow-up scenarios

To make DCE results more practical for policy makers, we calculated choice probabilities for several scenarios. To simulate current practice, the alternative ‘current intensive (and expensive) BCC follow-up practice’ was fixed at the following attribute levels: standard post-treatment visits performed by a different person treatment provider, extra information provided by a general handout, two additional follow-up visits in a year conducted by a dermatologist and a ten minute consultation during which the face, upper body and treated area is checked. The choice probabilities were calculated with different scenarios of ‘no BCC follow-up according to guideline’, to determine the optimal choice probability. The scenarios are based on previous qualitative studies [9, 10]. The expected choice probability scenarios are calculated on LCM results, by which the exponent of the total utility for a certain alternative was taken and divided by the exponent of utility of all alternatives in that scenario taking the class probabilities into account. The ‘current intensive BCC follow-up practice’ and ‘no BCC follow-up according guideline’ were chosen to resemble real life situations to determine which scenario without low-value follow-up visits is the most acceptable scenario by patients.

## Results

### Respondents’ characteristics

A total of 266 patients (72% response rate) and 131 dermatologists (21% response rate) completed and returned the questionnaire. Twelve dermatologists (9.1%) and 49 BCC patients (18%) failed the consistency test), however excluding these respondents only affected the significance due to lower sample size, therefore all respondents were included in the final analysis. The characteristics of the BCC patients and dermatologists are displayed in Table 2.

**Table 2:** BCC patients’ and dermatologists’ characteristics.

	Patients (n = 266)	Dermatologists (n = 131)
**Age, *mean years (SD)***	67.2 (12.3)	42.9 (10.9)
**Male**	49.6%	36.6%
**Type of healthcare centre**		
** • University hospital**	32.7%	21.4%
** • General hospital**	35.7%	24.4%
** • ISTC**	31.6%	13.7%
** • Multiple types**	N/A	9.9%
** • Missing**	0	30.5%
**Educational level**		
** • Low**	29.3%	0%
** • Medium**	39.1%	0%
** • High**	28.2%	100%
** • Missing**	3.4%	0%
**Income^1^**		
** • Low**	17.3%	N/A
** • Medium**	27.8%	N/A
** • High**	46.2%	N/A
** • Missing**	8.6%	N/A
**History of skin cancer**		
** • BCC**	34.2%	N/A
** • Other type**	9.8%	N/A
** • None**	54.9%	N/A
** • Missing**	1.1%	N/A
**EQ-VAS score, *Mean (SD)***	80.9 (13.5)	N/A
**Occupation**		
** • Dermatologist**	N/A	76.3%
** • Dermatology resident**	N/A	22.9%
** • Missing**	N/A	0.8%
**Working experience (including residency) *Mean years (SD)***	N/A	13.8 (10.7)
**Subspecialisation in dermato-oncology**		
** • Yes**	N/A	26.9%
** • No**	N/A	32.7%
** • Missing**	N/A	40.4%

SD, standard deviation; ISTC, Independent sector treatment centre; BCC, Basal cell carcinoma; EQ-VAS, EuroQol visual analogue scale; N/A, not applicable.

^1^Low, medium and high income represents <€1.500, €1.500-€2.500 and >€2.500 net monthly personal income respectively.

### Latent class analysis of patients and dermatologists

#### BCC patients

Three latent classes of preference patterns were identified for the BCC patients, with class probabilities for class 1, 2 and 3 being 13%, 46% and 41% respectively (Table 3). The patients belonging to class 1 did not have a significant preference regarding receiving additional follow-up care. However, if they would receive additional follow-up care, they would strongly prefer this to be conducted by their general practitioner (GP). These were predominantly patients older than 65 years without a BCC in their medical history. The BCC patients belonging to class 2 had a strong preference to receive additional follow-up care. They preferred two additional follow-up visits at 6 and 12 months after treatment, conducted by a dermatologist and to have the standard post-treatment visit performed by the same person as the treatment provider. In addition, they would prefer to receive a personalised letter and preferred the follow up visits to include a total-body skin examination. The respondents with high probability to belong to this class were mainly women. The patients belonging to the third class strongly preferred no additional follow-up visits and if there would be additional follow-up, they would prefer the standard post-treatment visit performed by the same person as the treatment provider and the follow-up visit to last 15 minutes and to be conducted by their GP.

**Table 3:** Latent class analysis of BCC patients’ (n = 266) preferences regarding BCC follow-up care.

	Class 1			Class 2			Class 3		
	Co-efficient	s.e.	p-value	Co-efficient	s.e.	p-value	Co-efficient	s.e.	p-value
**With additional follow-up visit(s)**									
Standard post-treatment visit performed:									
Not by same person as treatment provider (Ref)	0.16			-0.27			-0.48		
By the same person as treatment provider (β1)	-0.16	1.655	0.92	0.27	0.043	<0.01	0.48	0.139	<0.01
In addition to oral information, extra information will be provided by:									
E-health (Ref)	-0.75			0.03			-0.09		
Personalised Letter (β2)	1.45	4.298	0.74	0.14	0.097	0.15	-0.13	0.343	0.71
General hand-out (β3)	0.00	1.384	>0.99	0.04	0.102	0.72	0.10	0.269	0.71
General website (β4)	-0.70	3.540	0.84	-0.21	0.108	0.05	0.12	0.318	0.69
The additional follow-up visit(s) will be planned:									
1 year after treatment (Ref)	1.50			-0.06			0.28		
6 and 12 months after treatment (β5)	-0.40	2.676	0.88	0.15	0.053	<0.01	0.08	0.201	0.70
1 and 2 years after treatment (β6)	-1.10	1.650	0.50	-0.09	0.068	0.18	-0.36	0.190	0.06
The additional follow-up visit(s) will be conducted by:									
Nurse practitioner (Ref)	-1.31			0.06			-0.58		
General practitioner (β7)	4.23	1.540	<0.01	-0.18	0.467	<0.01	1.31	0.154	<0.01
Dermatologist (β8)	-2.92	1.355	0.03	0.12	0.052	0.02	-0.73	0.240	<0.01
The duration of the additional follow-up visit(s) will be:									
5 minutes (Ref)	-1.16			-0.16			-0.26		
10 minutes (β9)	2.28	1.243	0.07	0.05	0.079	0.56	-0.17	0.208	0.41
15 minutes (β10)	-1.12	1.191	0.35	0.11	0.070	0.12	0.43	0.173	0.01
Part of skin to be checked during the additional follow-up visits:									
Face, upper body and treated area (Ref)	0.55			-0.11			-0.01		
Full body (β11)	-0.55	0.472	0.24	0.11	0.029	<0.01	0.01	0.152	0.94
**Without additional follow-up visit**									
Constant (no follow-up) (β12)	0.50	1.352	0.71	-2.15	0.115	<0.01	2.87	0.178	<0.01
Standard post-treatment visit performed:									
Not by same person as treatment provider (Ref)	-0.88			-0.59			0.13		
By the same person as treatment provider (β13)	0.88	0.770	0.25	0.59	0.148	<0.01	-0.13	0.235	0.59
In addition to oral information, extra information will be provided by:									
E-health (Ref)	-1.21			0.00			0.26		
Personalised Letter (β14)	1.20	3.045	0.69	0.00	0.241	>0.99	-0.13	0.235	0.59
General hand-out (β15)	-0.51	1.787	0.78	-0.23	0.286	0.43	-0.25	0.266	0.34
General website (β16)	0.52	3.487	0.88	0.23	0.247	0.36	0.12	0.329	0.72
**Class probability model**									
Constant	-2.23	1.405	0.11	0.77	0.340	0.02	-		
Gender (male)	0.09	0.786	0.91	-1.45	0.377	<0.01	-		
Older age (≥65 years)	1.82	1.074	0.09	0.04	0.372	0.91	-		
Medical history of BCC (yes)	-0.58	0.016	<0.01	0.00	0.000	0.79	-		
**Class probabilities (%)**									
Average	13			46			41		
**Log Likelihood:** -1675.151									

Effects coding was used to determine the effects of all attribute levels [32].

BCC, Basal cell carcinoma; s.e., standard error; Ref, Reference.

#### Choice probabilities of BCC follow-up alternatives

**Fig 2:**
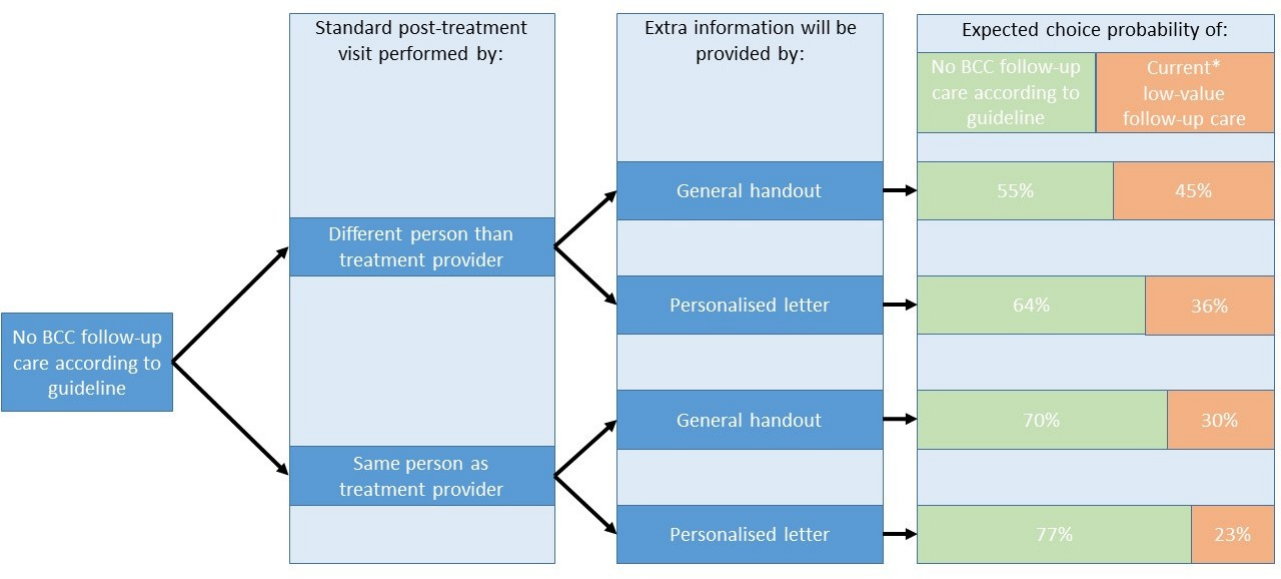
Expected patient choice probabilities of different BCC follow-up scenarios based on latent class model. *standard post-treatment visits performed by a different person than treatment provider, extra information provided by a general handout, two additional follow-up visits in a year conducted by a dermatologist and a ten minute consultation in which the face, upper body and treated area are checked.

The choice probability of ‘No BCC follow-up according to guideline’ was 55% if the standard post-treatment visit would not be performed by the same person as the treatment provider and if patients would receive a general hand-out compared to ‘Current intensive BCC follow-up’. This choice probability of ‘No BCC follow-up according to guideline’ increased from 55% up to 77%, if the standard post-treatment visit would be performed by the same person as treatment provider and if patients were offered a personalised handout as additional information (Fig 2).

#### Dermatologists

Two classes could be identified in the LCA regarding dermatologists (Table 4). The dermatologists belonging to class 1 strongly preferred no additional follow-up visits (i.e. according to the guideline). This contrasts with the dermatologists belonging to class 2, who strongly preferred additional follow-up visits, which they would prefer to be conducted by a dermatologist and include a total-body skin examination. The average class probabilities were 71% for class 1 and 29% for class 2. No covariates could significantly explain the latent class probabilities for dermatologists.

**Table 4:** Latent class analysis of dermatologists’ (n = 131) preferences regarding BCC follow-up care.

	Class 1			Class 2		
	Co-efficient	s.e.	p-value	Co-efficient	s.e.	p-value
**With additional follow-up visit(s)**						
Standard post-treatment visit performed:						
Not by same person as treatment provider (Ref)	-0.94			-0.11		
By the same person as treatment provider (β1)	0.94	0.210	<0.01	0.11	0.125	0.38
In addition to oral information, extra information will be provided by:						
E-health (Ref)	0.30			0.19		
Personalised Letter (β2)	-0.32	0.337	0.34	-0.16	0.232	0.49
General hand-out (β3)	0.17	0.263	0.52	0.23	0.212	0.28
General website (β4)	-0.15	0.269	0.57	-0.26	0.211	0.21
The additional follow-up visit(s) will be planned:						
1 year after treatment (Ref)	0.75			0.48		
6 and 12 months after treatment (β5)	-0.59	0.292	0.04	-0.04	0.153	0.82
1 and 2 years after treatment (β6)	-0.16	0.264	0.55	-0.44	0.168	<0.01
The additional follow-up visit(s) will be conducted by:						
Nurse practitioner (Ref)	-0.31			0.13		
General practitioner (β7)	0.13	0.233	0.58	-0.91	0.184	<0.01
Dermatologist (β8)	0.18	0.253	0.47	0.78	0.184	<0.01
The duration of the additional follow-up visit(s) will be:						
5 minutes (Ref)	-0.12			0.18		
10 minutes (β9)	-0.18	0.268	0.51	-0.06	0.20	0.76
15 minutes (β10)	0.30	0.252	0.23	-0.12	0.16	0.44
Part of skin to be checked during the additional follow-up visits:						
Face, upper body and treated area (Ref)	0.06			-0.37		
Full body (β11)	-0.06	0.167	0.70	0.37	0.106	<0.01
**Without additional follow-up visit**						
Constant (no follow-up) (β12)	3.29	0.267	<0.01	-1.81	0.370	<0.01
Standard post-treatment visit performed:						
Not by same person as treatment provider (Ref)	-0.92			-0.58		
By the same person as treatment provider (β13)	0.92	0.201	<0.01	0.58	0.317	0.07
In addition to oral information, extra information will be provided by:						
E-health (Ref)	-0.36			0.72		
Personalised Letter (β14)	0.09	0.306	0.76	0.00	0.567	>0.99
General hand-out (β15)	0.22	0.294	0.46	-0.87	0.809	0.28
General website (β16)	0.05	0.328	0.88	0.15	0.519	0.78
**Class probabilities (%)**						
Average	71%			29%		
**Log likelihood:** -306.016						

Effects coding was used to determine the effects of all attribute levels [32].

BCC, Basal cell carcinoma; s.e., standard error; Ref, Reference.

### Multinomial logit with interaction terms of dermatologists

To test for interaction, the covariates age over 40 years, male gender and subspecialisation in oncology were multiplied with the strongest identifier of the classes: ‘no additional follow up’. This model demonstrated that predominantly young and male dermatologists preferred no additional follow-up for BCC patients (p = <0.01). Having a subspecialisation in oncology did not affect the preference for this attribute.

## Discussion

The current study quantified the preferences and needs of patients and dermatologists to accept a reduction of low-risk BCC follow-up care. We found that a reduction would be more acceptable if the post-treatment visit were to be performed by the same person as the treatment provider and additional information would be provided by a personalised letter for patients.

The observation of a preference for personalised letter as a feasible option to reduce low-value follow-up visits is supported by the preference for personalised information which was previously expressed in focus groups with BCC patients [9]. Other studies have also shown that personalised information satisfies cancer patients more than general information, as this tells them something new and is more often relevant to them [33]. A preference for continuity of care (e.g. same healthcare provider for treatment as post-treatment visit) has also been well documented to have positive effects on indicators of quality of care, such as increased patient satisfaction, decreased hospitalisations, decreased emergency department visits and improved receipt of preventive services [34].

Mainly female patients preferred additional low-value BCC follow-up visits, conducted by a dermatologist. In contrast, predominantly male patients preferred to not receive additional follow-up, and should they get follow-up, they preferred the GP to conduct this. In contrast to studies where patients with other types of cancer preferred their follow-up care to be conducted by a medical specialist [15, 20], overall, the patients in our study preferred their GP for their follow-up care. This could be explained by the inclusion of the out-of-pocket costs in our DCE to make our scenarios as realistic as possible, which was linked to ‘type of healthcare professional’ and ‘frequency of follow-up’. In addition, recent focus groups with low-risk BCC patients showed that they perceived their BCC as a not severe type of cancer and some patients actually preferred their GP to conduct follow-up care as he or she is closer to home and easier approachable [10].

A limitation in this study is the low response rate of dermatologists. Although response rates are generally low among medical specialists (27%-68%) [26–29], the 21% in the current study was even lower. This could be explained by the facts that the DCE invitation was sent through a non-personal newsletter and that it encompassed a lengthy questionnaire. Additionally, we could not offer the physicians compensation and reminders could not be sent as they responded anonymously. This low participation may have led to a selection bias, as reflected in the mean age of the respondents being 43 years, which is lower than the nationwide mean age of dermatologists and residents [35]. The current study revealed that young male dermatologists preferred no additional follow-up care, which is in accordance with influencing factors explored in interviews with dermatologists [10]. Inclusion of older dermatologists would probably shift the results to more dermatologists preferring additional follow-up care, which would emphasize the need for a de-adoption strategy.

In conclusion, these results form a solid foundation for a feasible strategy to reduce the low-value BCC follow-up care, while maintaining quality of care. This follow-up would consist of having one healthcare provider for the initial treatment as well as the standard post-treatment evaluation combined with a personalised letter for the patient with information about their diagnosis, received treatment and personalised follow-up schedule. Further research is needed to determine whether implementation of this intervention will decrease the amount of low-value follow-up visits in practice, while maintaining patient satisfaction.

## Supporting information

S1 Appendix (DOCX)

S2 Appendix (DOCX)
