# Supplementary material for: What do patients and dermatologists prefer regarding low-risk basal cell carcinoma follow-up care? A discrete choice experiment
Source: PLoS One. 2021 Mar 29;16(3):e0249298. doi: 10.1371/journal.pone.0249298 (PMC8007023; doi:10.1371/journal.pone.0249298)
Supplement: S1 Appendix — (DOCX) [file pone.0249298.s001.docx]

**Discrete choice experiment**

Explanation of the choice situations: In the following questions you always have the choice of 3 fictional choice situations: follow-up scenario A, follow-up scenario B and follow-up scenario C. You will notice that the choice situations are similar, yet they are always slightly different. There are no right or wrong answers. Each time, we want you to choose which of the three choice situations you prefer.

The current guideline for follow-up: The type of skin cancer basal cell carcinoma (BCC) has a good prognosis. A BCC grows slowly, almost never metastasizes, and is easily curable. The current guideline states that a patient with 1 BCC, which has been treated with good results, does not need to come back for further follow-up visits. After treatment there is a standard visit, the practitioner checks then whether the skin cancer has been properly treated. In addition, you will receive explanation about the skin cancer and how you can examine your own skin. Should you discover a suspicious skin abnormality in the future, you can visit for an appointment within a short time in an easily accessible way.

'Additional information': We would like to know in what way you, in addition to the oral explanation, would like to receive additional information about the severity of the treated skin cancer, what the expectations are regarding the skin cancer, further treatment and/or follow-up and the explanation you will receive on how you can examine your own skin. This can be general information or tailored to your specific situation. With e-health, customised information is given via the Internet.

‘The extra follow-up check is carried out by’: the dermatologist, general practitioner or nurse practitioner. The general practitioners and nurse practitioners in these options received additional training to perform the follow-up visits. A dermatology nurse practitioner is specialised in the field of skin cancer with additional medical training. He/she may independently treat and monitor skin cancer patients and may consult with the dermatologist if necessary.

Costs: In the follow-up visit schedules, there is an amount indicated that you must pay in total yourself from your deductible. Follow-up care by the dermatologist costs €115 per year, follow-up care by the nurse specialist costs €85 per year, and follow-up care by the general practitioner falls outside the "excess" amount and therefore costs €0 per year.

Skin examination: According to the guideline, it is recommended to examine the treated area and the skin exposed to sunlight as a whole. The skin of the face, upper body and arms are the most exposed to the sun, so these parts have the highest risk of developing a BCC. Also the treated part of the skin has a higher risk of e.g. BCC recurrence. In addition to these higher-risk areas, the other skin areas are also at risk of developing BCCs.

We ask that you check the box under the choice situation that best suits your preference for the following questions.

- Imagine you have basal cell carcinoma for the first time and have been fully treated for it, what would be your preference for post-treatment follow-up?

| **Question 1** | **Follow-up scenario A** | **Follow-up scenario B** | **Follow-up scenario C** |
| --- | --- | --- | --- |
| Standard post-treatment visit performed: | By the same person as treatment provider | By the same person as treatment provider | By the same person as treatment provider |
| In addition to oral information, extra information will be provided by: | Personalised letter | General hand-out | Personalised letter |
| The additional follow-up visit(s) will be planned: | There will be no additional follow-up visit planned. The patient will make an appointment if he or she finds a suspicious lesion. | 1 year after treatment | 6 months and 1 year after treatment |
| The additional follow-up visit(s) will be conducted by: | N/A  (Self-examination) | General practitioner | Dermatologist |
| The out-of-pocket costs for this follow-up scenario will be: | 0 euro | 0 euro | 115 euro |
| The duration of the additional follow-up visit(s) will be: | N/A  (Self-examination) | 5 minutes | 15 minutes |
| Part of skin to be checked during the additional follow-up visits: | N/A  (Self-examination) | Face, upper body and treated area | Full body |
|  | 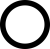 | 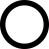 | 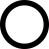 |

| **Question 2** | **Follow-up scenario A** | **Follow-up scenario B** | **Follow-up scenario C** |
| --- | --- | --- | --- |
| Standard post-treatment visit performed: | By the same person as treatment provider | Not by same person as treatment provider | By the same person as treatment provider |
| In addition to oral information, extra information will be provided by: | Personalised letter | General hand-out | General website |
| The additional follow-up visit(s) will be planned: | There will be no additional follow-up visit planned. The patient will make an appointment if he or she finds a suspicious lesion. | 1 and 2 years after treatment | 1 year after treatment |
| The additional follow-up visit(s) will be conducted by: | N/A  (Self-examination) | Dermatologist | Nurse practitioner |
| The out-of-pocket costs for this follow-up scenario will be: | 0 euro | 230 euro | 85 euro |
| The duration of the additional follow-up visit(s) will be: | N/A  (Self-examination) | 15 minutes | 10 minutes |
| Part of skin to be checked during the additional follow-up visits: | N/A  (Self-examination) | Full body | Face, upper body and treated area |
|  | 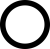 | 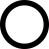 | 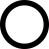 |

| **Question 3** | **Follow-up scenario A** | **Follow-up scenario B** | **Follow-up scenario C** |
| --- | --- | --- | --- |
| Standard post-treatment visit performed: | Not by same person as treatment provider | By the same person as treatment provider | Not by same person as treatment provider |
| In addition to oral information, extra information will be provided by: | General website | General hand-out | E-health |
| The additional follow-up visit(s) will be planned: | There will be no additional follow-up visit planned. The patient will make an appointment if he or she finds a suspicious lesion. | 1 and 2 years after treatment | 1 year after treatment |
| The additional follow-up visit(s) will be conducted by: | N/A  (Self-examination) | Nurse practitioner | General practitioner |
| The out-of-pocket costs for this follow-up scenario will be: | 0 euro | 170 euro | 0 euro |
| The duration of the additional follow-up visit(s) will be: | N/A (Self-examination) | 15 minutes | 10 minutes |
| Part of skin to be checked during the additional follow-up visits: | N/A (Self-examination) | Full body | Face, upper body and treated area |
|  | 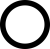 | 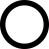 | 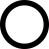 |

| **Question 4** | **Follow-up scenario A** | **Follow-up scenario B** | **Follow-up scenario C** |
| --- | --- | --- | --- |
| Standard post-treatment visit performed: | By the same person as treatment provider | By the same person as treatment provider | Not by same person as treatment provider |
| In addition to oral information, extra information will be provided by: | Personalised letter | E-health | Personalised letter |
| The additional follow-up visit(s) will be planned: | There will be no additional follow-up visit planned. The patient will make an appointment if he or she finds a suspicious lesion. | 6 months and 1 year after treatment | 1 and 2 years after treatment |
| The additional follow-up visit(s) will be conducted by: | N/A  (Self-examination) | General practitioner | Dermatologist |
| The out-of-pocket costs for this follow-up scenario will be: | 0 euro | 0 euro | 230 euro |
| The duration of the additional follow-up visit(s) will be: | N/A (Self-examination) | 10 minutes | 5 minutes |
| Part of skin to be checked during the additional follow-up visits: | N/A (Self-examination) | Full body | Face, upper body and treated area |
|  | 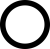 | 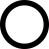 | 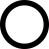 |

| **Question 5** | **Follow-up scenario A** | **Follow-up scenario B** | **Follow-up scenario C** |
| --- | --- | --- | --- |
| Standard post-treatment visit performed: | Not by same person as treatment provider | By the same person as treatment provider | Not by same person as treatment provider |
| In addition to oral information, extra information will be provided by: | E-health | Personalised letter | General hand-out |
| The additional follow-up visit(s) will be planned: | There will be no additional follow-up visit planned. The patient will make an appointment if he or she finds a suspicious lesion. | 1 year after treatment | 1 and 2 years after treatment |
| The additional follow-up visit(s) will be conducted by: | N/A  (Self-examination) | Dermatologist | General practitioner |
| The out-of-pocket costs for this follow-up scenario will be: | 0 euro | 115 euro | 0 euro |
| The duration of the additional follow-up visit(s) will be: | N/A (Self-examination) | 5 minutes | 15 minutes |
| Part of skin to be checked during the additional follow-up visits: | N/A (Self-examination) | Face, upper body and treated area | Full body |
|  | 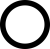 | 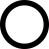 | 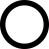 |

| **Question 6** | **Follow-up scenario A** | **Follow-up scenario B** | **Follow-up scenario C** |
| --- | --- | --- | --- |
| Standard post-treatment visit performed: | By the same person as treatment provider | By the same person as treatment provider | Not by same person as treatment provider |
| In addition to oral information, extra information will be provided by: | E-health | General website | E-health |
| The additional follow-up visit(s) will be planned: | There will be no additional follow-up visit planned. The patient will make an appointment if he or she finds a suspicious lesion. | 1 and 2 years after treatment | 6 months and 1 year after treatment |
| The additional follow-up visit(s) will be conducted by: | N/A  (Self-examination) | Dermatologist | Nurse practitioner |
| The out-of-pocket costs for this follow-up scenario will be: | 0 euro | 230 euro | 85 euro |
| The duration of the additional follow-up visit(s) will be: | N/A (Self-examination) | 15 minutes | 10 minutes |
| Part of skin to be checked during the additional follow-up visits: | N/A (Self-examination) | Face, upper body and treated area | Full body |
|  | 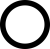 | 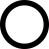 | 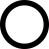 |

| **Question 7** | **Follow-up scenario A** | **Follow-up scenario B** | **Follow-up scenario C** |
| --- | --- | --- | --- |
| Standard post-treatment visit performed: | Not by same person as treatment provider | Not by same person as treatment provider | By the same person as treatment provider |
| In addition to oral information, extra information will be provided by: | General hand-out | E-health | General hand-out |
| The additional follow-up visit(s) will be planned: | There will be no additional follow-up visit planned. The patient will make an appointment if he or she finds a suspicious lesion. | 6 months and 1 year after treatment | 1 and 2 years after treatment |
| The additional follow-up visit(s) will be conducted by: | N/A  (Self-examination) | Dermatologist | General practitioner |
| The out-of-pocket costs for this follow-up scenario will be: | 0 euro | 115 euro | 0 euro |
| The duration of the additional follow-up visit(s) will be: | N/A (Self-examination) | 15 minutes | 5 minutes |
| Part of skin to be checked during the additional follow-up visits: | N/A (Self-examination) | Face, upper body and treated area | Full body |
|  | 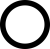 | 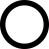 | 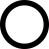 |

| **Question 8** | **Follow-up scenario A** | **Follow-up scenario B** | **Follow-up scenario C** |
| --- | --- | --- | --- |
| Standard post-treatment visit performed: | Not by same person as treatment provider | Not by same person as treatment provider | By the same person as treatment provider |
| In addition to oral information, extra information will be provided by: | General website | General website | General hand-out |
| The additional follow-up visit(s) will be planned: | There will be no additional follow-up visit planned. The patient will make an appointment if he or she finds a suspicious lesion. | 1 year after treatment | 6 months and 1 year after treatment |
| The additional follow-up visit(s) will be conducted by: | N/A  (Self-examination) | General practitioner | Nurse practitioner |
| The out-of-pocket costs for this follow-up scenario will be: | 0 euro | 0 euro | 85 euro |
| The duration of the additional follow-up visit(s) will be: | N/A (Self-examination) | 10 minutes | 5 minutes |
| Part of skin to be checked during the additional follow-up visits: | N/A (Self-examination) | Full body | Face, upper body and treated area |
|  | 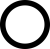 | 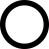 | 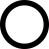 |

| **Question 9** | **Follow-up scenario A** | **Follow-up scenario B** | **Follow-up scenario C** |
| --- | --- | --- | --- |
| Standard post-treatment visit performed: | Not by same person as treatment provider | By the same person as treatment provider | Not by same person as treatment provider |
| In addition to oral information, extra information will be provided by: | General hand-out | E-health | General hand-out |
| The additional follow-up visit(s) will be planned: | There will be no additional follow-up visit planned. The patient will make an appointment if he or she finds a suspicious lesion. | 1 and 2 years after treatment | 1 year after treatment |
| The additional follow-up visit(s) will be conducted by: | N/A  (Self-examination) | General practitioner | Dermatologist |
| The out-of-pocket costs for this follow-up scenario will be: | 0 euro | 0 euro | 115 euro |
| The duration of the additional follow-up visit(s) will be: | N/A (Self-examination) | 10 minutes | 5 minutes |
| Part of skin to be checked during the additional follow-up visits: | N/A (Self-examination) | Full body | Face, upper body and treated area |
|  | 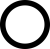 | 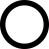 | 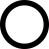 |

| **Question 10** | **Follow-up scenario A** | **Follow-up scenario B** | **Follow-up scenario C** |
| --- | --- | --- | --- |
| Standard post-treatment visit performed: | By the same person as treatment provider | By the same person as treatment provider | Not by same person as treatment provider |
| In addition to oral information, extra information will be provided by: | General hand-out | Personalised letter | General website |
| The additional follow-up visit(s) will be planned: | There will be no additional follow-up visit planned. The patient will make an appointment if he or she finds a suspicious lesion. | 6 months and 1 year after treatment | 1 year after treatment |
| The additional follow-up visit(s) will be conducted by: | N/A  (Self-examination) | Nurse practitioner | Dermatologist |
| The out-of-pocket costs for this follow-up scenario will be: | 0 euro | 85 euro | 115 euro |
| The duration of the additional follow-up visit(s) will be: | N/A (Self-examination) | 5 minutes | 10 minutes |
| Part of skin to be checked during the additional follow-up visits: | N/A (Self-examination) | Face, upper body and treated area | Full body |
|  | 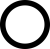 | 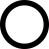 | 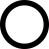 |

| **Question 11** | **Follow-up scenario A** | **Follow-up scenario B** | **Follow-up scenario C** |
| --- | --- | --- | --- |
| Standard post-treatment visit performed: | Not by same person as treatment provider | Not by same person as treatment provider | By the same person as treatment provider |
| In addition to oral information, extra information will be provided by: | Personalised letter | Personalised letter | E-health |
| The additional follow-up visit(s) will be planned: | There will be no additional follow-up visit planned. The patient will make an appointment if he or she finds a suspicious lesion. | 1 year after treatment | 6 months and 1 year after treatment |
| The additional follow-up visit(s) will be conducted by: | N/A  (Self-examination) | General practitioner | Nurse practitioner |
| The out-of-pocket costs for this follow-up scenario will be: | 0 euro | 0 euro | 85 euro |
| The duration of the additional follow-up visit(s) will be: | N/A (Self-examination) | 10 minutes | 15 minutes |
| Part of skin to be checked during the additional follow-up visits: | N/A (Self-examination) | Face, upper body and treated area | Full body |
|  | 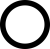 | 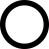 | 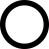 |

| **Question 12** | **Follow-up scenario A** | **Follow-up scenario B** | **Follow-up scenario C** |
| --- | --- | --- | --- |
| Standard post-treatment visit performed: | By the same person as treatment provider | Not by same person as treatment provider | By the same person as treatment provider |
| In addition to oral information, extra information will be provided by: | E-health | Personalised letter | E-health |
| The additional follow-up visit(s) will be planned: | There will be no additional follow-up visit planned. The patient will make an appointment if he or she finds a suspicious lesion. | 6 months and 1 year after treatment | 1 and 2 years after treatment |
| The additional follow-up visit(s) will be conducted by: | N/A  (Self-examination) | Dermatologist | General practitioner |
| The out-of-pocket costs for this follow-up scenario will be: | 0 euro | 115 euro | 0 euro |
| The duration of the additional follow-up visit(s) will be: | N/A (Self-examination) | 10 minutes | 5 minutes |
| Part of skin to be checked during the additional follow-up visits: | N/A (Self-examination) | Full body | Face, upper body and treated area |
|  | 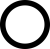 | 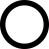 | 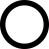 |

| **Question 13** | **Follow-up scenario A** | **Follow-up scenario B** | **Follow-up scenario C** |
| --- | --- | --- | --- |
| Standard post-treatment visit performed: | By the same person as treatment provider | By the same person as treatment provider | Not by same person as treatment provider |
| In addition to oral information, extra information will be provided by: | Personalised letter | E-health | Personalised letter |
| The additional follow-up visit(s) will be planned: | There will be no additional follow-up visit planned. The patient will make an appointment if he or she finds a suspicious lesion. | 6 months and 1 year after treatment | 1 and 2 years after treatment |
| The additional follow-up visit(s) will be conducted by: | N/A  (Self-examination) | General practitioner | Dermatologist |
| The out-of-pocket costs for this follow-up scenario will be: | 0 euro | 0 euro | 230 euro |
| The duration of the additional follow-up visit(s) will be: | N/A (Self-examination) | 10 minutes | 5 minutes |
| Part of skin to be checked during the additional follow-up visits: | N/A (Self-examination) | Full body | Face, upper body and treated area |
|  | 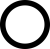 | 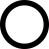 | 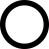 |
