# Supplementary material for: What do patients and dermatologists prefer regarding low-risk basal cell carcinoma follow-up care? A discrete choice experiment
Source: PLoS One. 2021 Mar 29;16(3):e0249298. doi: 10.1371/journal.pone.0249298 (PMC8007023; doi:10.1371/journal.pone.0249298)
Supplement: S2 Appendix — (DOCX) [file pone.0249298.s002.docx]

**Discrete choice experiment**

In the following 25 questions, you will repeatedly have a choice between 3 fictional situations: follow-up scenario A, follow-up scenario B, and follow-up scenario C. The situations are intentionally similar, but slightly different each time. It is important to answer all of the choice situations in order to mathematically determine preferences. There are no right or wrong answers. Each time, we want you to choose which of the 3 choice situations you prefer.

Current BCC follow-up guideline:

"It is worth considering monitoring patients after BCCs with high risk of local recurrence such as large and aggressively growing primary BCCs and recurrent BCCs on the face where a (subsequent) recurrence could cause a high degree of morbidity.

The study group recommends that after treatment without histologic monitoring for radicality, at least one check should be performed 6-12 months after treatment.

The workgroup recommends that all patients be instructed on self-examination and that patients be given a BCC information leaflet or pointed to the digital BCC information leaflet, preferably the one from the NVDV website."

|  | **Low-risk** | **High-risk** |
| --- | --- | --- |
| **Histological growth type** | Non-aggressive (nodular, superficial) | Aggressive (infiltrative, micronodular) |
| **Localisation** | Trunk | H-zone (yes, ears, lips, nasolabial fold, nose) |
| **Size** | < 2 cm | ≥ 2 cm |
| **Previous therapy** | Primaire tumor | Recidief tumor |

With these questions we would like to explore your preferences when it comes to the follow-up care of patients after the treatment of a primary low-risk BCC.

Additional information to the items:

| **Standard post-treatment visit performed:** | The follow-up check can be performed by the treatment provider, or by another person. |
| --- | --- |
| **In addition to oral information, extra information will be provided by:** | Patients may wish to receive additional information, in addition to the verbal explanation, about the severity of the treated BCC, about the prognosis, further treatment and/or follow-up monitoring, and explanations about how they can examine their own skin. This can be general information or tailored to the patient's specific situation. With e-health, customized information is given via the Internet. |
| **The additional follow-up visit(s) will be planned:** | The patient comes back immediately after the treatment as standard once for check-up of the treatment, with additional follow-up control is meant the follow-up visit after e.g. after 1 year. |
| **The additional follow-up visit(s) will be conducted by:** | The options are: dermatologist, general practitioner or nurse practitioner. The general practitioners and nurse practitioners in these options received additional training to conduct the follow-up. A nurse practitioner in dermatology is specialized in the field of skin cancer with additional medical training. He/she may independently treat and monitor skin cancer patients and may consult the dermatologist if necessary. |
| **The out-of-pocket costs for this follow-up scenario will be:** | In the follow-up schedules, there is an amount indicated that the patient must pay in total him/herself from the deductible. Follow-up by the dermatologist costs €115 per year, by the nurse practitioner costs €85 per year and follow-up by the general practitioner falls outside the 'deductible' amount and thus costs €0 per year. |
| **The duration of the additional follow-up visit(s) will be:** | The consultation time for the additional follow-up visits can vary between no follow-up (self-examination), 5 minutes, 10 minutes or 15 minutes. |
| **Part of skin to be checked during the additional follow-up visits:** | According to the guideline, careful inspection of the scar and control of the entire skin is advised |

| **Question 1** | **Follow-up scenario A** | **Follow-up scenario B** | **Follow-up scenario C** |
| --- | --- | --- | --- |
| Standard post-treatment visit performed: | By the same person as treatment provider | By the same person as treatment provider | By the same person as treatment provider |
| In addition to oral information, extra information will be provided by: | Personalised letter | General hand-out | Personalised letter |
| The additional follow-up visit(s) will be planned: | There will be no additional follow-up visit planned. The patient will make an appointment if he or she finds a suspicious lesion. | 1 year after treatment | 6 months and 1 year after treatment |
| The additional follow-up visit(s) will be conducted by: | N/A  (Self-examination) | General practitioner | Dermatologist |
| The out-of-pocket costs for this follow-up scenario will be: | 0 euro | 0 euro | 115 euro |
| The duration of the additional follow-up visit(s) will be: | N/A  (Self-examination) | 5 minutes | 15 minutes |
| Part of skin to be checked during the additional follow-up visits: | N/A  (Self-examination) | Face, upper body and treated area | Full body |
|  | 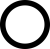 | 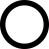 | 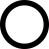 |

| **Question 2** | **Follow-up scenario A** | **Follow-up scenario B** | **Follow-up scenario C** |
| --- | --- | --- | --- |
| Standard post-treatment visit performed: | By the same person as treatment provider | Not by same person as treatment provider | By the same person as treatment provider |
| In addition to oral information, extra information will be provided by: | Personalised letter | General hand-out | General website |
| The additional follow-up visit(s) will be planned: | There will be no additional follow-up visit planned. The patient will make an appointment if he or she finds a suspicious lesion. | 1 and 2 years after treatment | 1 year after treatment |
| The additional follow-up visit(s) will be conducted by: | N/A  (Self-examination) | Dermatologist | Nurse practitioner |
| The out-of-pocket costs for this follow-up scenario will be: | 0 euro | 230 euro | 85 euro |
| The duration of the additional follow-up visit(s) will be: | N/A  (Self-examination) | 15 minutes | 10 minutes |
| Part of skin to be checked during the additional follow-up visits: | N/A  (Self-examination) | Full body | Face, upper body and treated area |
|  | 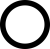 | 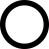 | 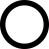 |

| **Question 3** | **Follow-up scenario A** | **Follow-up scenario B** | **Follow-up scenario C** |
| --- | --- | --- | --- |
| Standard post-treatment visit performed: | Not by same person as treatment provider | By the same person as treatment provider | Not by same person as treatment provider |
| In addition to oral information, extra information will be provided by: | General website | General hand-out | E-health |
| The additional follow-up visit(s) will be planned: | There will be no additional follow-up visit planned. The patient will make an appointment if he or she finds a suspicious lesion. | 1 and 2 years after treatment | 1 year after treatment |
| The additional follow-up visit(s) will be conducted by: | N/A  (Self-examination) | Nurse practitioner | General practitioner |
| The out-of-pocket costs for this follow-up scenario will be: | 0 euro | 170 euro | 0 euro |
| The duration of the additional follow-up visit(s) will be: | N/A (Self-examination) | 15 minutes | 10 minutes |
| Part of skin to be checked during the additional follow-up visits: | N/A (Self-examination) | Full body | Face, upper body and treated area |
|  | 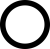 | 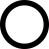 | 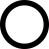 |

| **Question 4** | **Follow-up scenario A** | **Follow-up scenario B** | **Follow-up scenario C** |
| --- | --- | --- | --- |
| Standard post-treatment visit performed: | By the same person as treatment provider | By the same person as treatment provider | Not by same person as treatment provider |
| In addition to oral information, extra information will be provided by: | Personalised letter | E-health | Personalised letter |
| The additional follow-up visit(s) will be planned: | There will be no additional follow-up visit planned. The patient will make an appointment if he or she finds a suspicious lesion. | 6 months and 1 year after treatment | 1 and 2 years after treatment |
| The additional follow-up visit(s) will be conducted by: | N/A  (Self-examination) | General practitioner | Dermatologist |
| The out-of-pocket costs for this follow-up scenario will be: | 0 euro | 0 euro | 230 euro |
| The duration of the additional follow-up visit(s) will be: | N/A (Self-examination) | 10 minutes | 5 minutes |
| Part of skin to be checked during the additional follow-up visits: | N/A (Self-examination) | Full body | Face, upper body and treated area |
|  | 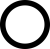 | 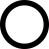 | 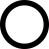 |

| **Question 5** | **Follow-up scenario A** | **Follow-up scenario B** | **Follow-up scenario C** |
| --- | --- | --- | --- |
| Standard post-treatment visit performed: | Not by same person as treatment provider | By the same person as treatment provider | Not by same person as treatment provider |
| In addition to oral information, extra information will be provided by: | E-health | Personalised letter | General hand-out |
| The additional follow-up visit(s) will be planned: | There will be no additional follow-up visit planned. The patient will make an appointment if he or she finds a suspicious lesion. | 1 year after treatment | 1 and 2 years after treatment |
| The additional follow-up visit(s) will be conducted by: | N/A  (Self-examination) | Dermatologist | General practitioner |
| The out-of-pocket costs for this follow-up scenario will be: | 0 euro | 115 euro | 0 euro |
| The duration of the additional follow-up visit(s) will be: | N/A (Self-examination) | 5 minutes | 15 minutes |
| Part of skin to be checked during the additional follow-up visits: | N/A (Self-examination) | Face, upper body and treated area | Full body |
|  | 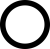 | 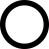 | 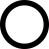 |

| **Question 6** | **Follow-up scenario A** | **Follow-up scenario B** | **Follow-up scenario C** |
| --- | --- | --- | --- |
| Standard post-treatment visit performed: | By the same person as treatment provider | By the same person as treatment provider | Not by same person as treatment provider |
| In addition to oral information, extra information will be provided by: | E-health | General website | E-health |
| The additional follow-up visit(s) will be planned: | There will be no additional follow-up visit planned. The patient will make an appointment if he or she finds a suspicious lesion. | 1 and 2 years after treatment | 6 months and 1 year after treatment |
| The additional follow-up visit(s) will be conducted by: | N/A  (Self-examination) | Dermatologist | Nurse practitioner |
| The out-of-pocket costs for this follow-up scenario will be: | 0 euro | 230 euro | 85 euro |
| The duration of the additional follow-up visit(s) will be: | N/A (Self-examination) | 15 minutes | 10 minutes |
| Part of skin to be checked during the additional follow-up visits: | N/A (Self-examination) | Face, upper body and treated area | Full body |
|  | 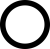 | 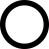 | 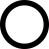 |

| **Question 7** | **Follow-up scenario A** | **Follow-up scenario B** | **Follow-up scenario C** |
| --- | --- | --- | --- |
| Standard post-treatment visit performed: | Not by same person as treatment provider | Not by same person as treatment provider | By the same person as treatment provider |
| In addition to oral information, extra information will be provided by: | General hand-out | E-health | General hand-out |
| The additional follow-up visit(s) will be planned: | There will be no additional follow-up visit planned. The patient will make an appointment if he or she finds a suspicious lesion. | 6 months and 1 year after treatment | 1 and 2 years after treatment |
| The additional follow-up visit(s) will be conducted by: | N/A  (Self-examination) | Dermatologist | General practitioner |
| The out-of-pocket costs for this follow-up scenario will be: | 0 euro | 115 euro | 0 euro |
| The duration of the additional follow-up visit(s) will be: | N/A (Self-examination) | 15 minutes | 5 minutes |
| Part of skin to be checked during the additional follow-up visits: | N/A (Self-examination) | Face, upper body and treated area | Full body |
|  | 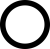 | 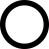 | 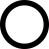 |

| **Question 8** | **Follow-up scenario A** | **Follow-up scenario B** | **Follow-up scenario C** |
| --- | --- | --- | --- |
| Standard post-treatment visit performed: | Not by same person as treatment provider | Not by same person as treatment provider | By the same person as treatment provider |
| In addition to oral information, extra information will be provided by: | General website | General website | General hand-out |
| The additional follow-up visit(s) will be planned: | There will be no additional follow-up visit planned. The patient will make an appointment if he or she finds a suspicious lesion. | 1 year after treatment | 6 months and 1 year after treatment |
| The additional follow-up visit(s) will be conducted by: | N/A  (Self-examination) | General practitioner | Nurse practitioner |
| The out-of-pocket costs for this follow-up scenario will be: | 0 euro | 0 euro | 85 euro |
| The duration of the additional follow-up visit(s) will be: | N/A (Self-examination) | 10 minutes | 5 minutes |
| Part of skin to be checked during the additional follow-up visits: | N/A (Self-examination) | Full body | Face, upper body and treated area |
|  | 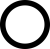 | 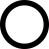 | 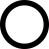 |

| **Question 9** | **Follow-up scenario A** | **Follow-up scenario B** | **Follow-up scenario C** |
| --- | --- | --- | --- |
| Standard post-treatment visit performed: | Not by same person as treatment provider | By the same person as treatment provider | Not by same person as treatment provider |
| In addition to oral information, extra information will be provided by: | General hand-out | E-health | General hand-out |
| The additional follow-up visit(s) will be planned: | There will be no additional follow-up visit planned. The patient will make an appointment if he or she finds a suspicious lesion. | 1 and 2 years after treatment | 1 year after treatment |
| The additional follow-up visit(s) will be conducted by: | N/A  (Self-examination) | General practitioner | Dermatologist |
| The out-of-pocket costs for this follow-up scenario will be: | 0 euro | 0 euro | 115 euro |
| The duration of the additional follow-up visit(s) will be: | N/A (Self-examination) | 10 minutes | 5 minutes |
| Part of skin to be checked during the additional follow-up visits: | N/A (Self-examination) | Full body | Face, upper body and treated area |
|  | 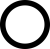 | 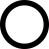 | 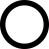 |

| **Question 10** | **Follow-up scenario A** | **Follow-up scenario B** | **Follow-up scenario C** |
| --- | --- | --- | --- |
| Standard post-treatment visit performed: | By the same person as treatment provider | By the same person as treatment provider | Not by same person as treatment provider |
| In addition to oral information, extra information will be provided by: | General hand-out | Personalised letter | General website |
| The additional follow-up visit(s) will be planned: | There will be no additional follow-up visit planned. The patient will make an appointment if he or she finds a suspicious lesion. | 6 months and 1 year after treatment | 1 year after treatment |
| The additional follow-up visit(s) will be conducted by: | N/A  (Self-examination) | Nurse practitioner | Dermatologist |
| The out-of-pocket costs for this follow-up scenario will be: | 0 euro | 85 euro | 115 euro |
| The duration of the additional follow-up visit(s) will be: | N/A (Self-examination) | 5 minutes | 10 minutes |
| Part of skin to be checked during the additional follow-up visits: | N/A (Self-examination) | Face, upper body and treated area | Full body |
|  | 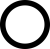 | 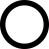 | 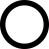 |

| **Question 11** | **Follow-up scenario A** | **Follow-up scenario B** | **Follow-up scenario C** |
| --- | --- | --- | --- |
| Standard post-treatment visit performed: | Not by same person as treatment provider | Not by same person as treatment provider | By the same person as treatment provider |
| In addition to oral information, extra information will be provided by: | Personalised letter | Personalised letter | E-health |
| The additional follow-up visit(s) will be planned: | There will be no additional follow-up visit planned. The patient will make an appointment if he or she finds a suspicious lesion. | 1 year after treatment | 6 months and 1 year after treatment |
| The additional follow-up visit(s) will be conducted by: | N/A  (Self-examination) | General practitioner | Nurse practitioner |
| The out-of-pocket costs for this follow-up scenario will be: | 0 euro | 0 euro | 85 euro |
| The duration of the additional follow-up visit(s) will be: | N/A (Self-examination) | 10 minutes | 15 minutes |
| Part of skin to be checked during the additional follow-up visits: | N/A (Self-examination) | Face, upper body and treated area | Full body |
|  | 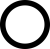 | 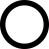 | 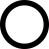 |

| **Question 12** | **Follow-up scenario A** | **Follow-up scenario B** | **Follow-up scenario C** |
| --- | --- | --- | --- |
| Standard post-treatment visit performed: | By the same person as treatment provider | Not by same person as treatment provider | By the same person as treatment provider |
| In addition to oral information, extra information will be provided by: | E-health | Personalised letter | E-health |
| The additional follow-up visit(s) will be planned: | There will be no additional follow-up visit planned. The patient will make an appointment if he or she finds a suspicious lesion. | 6 months and 1 year after treatment | 1 and 2 years after treatment |
| The additional follow-up visit(s) will be conducted by: | N/A  (Self-examination) | Dermatologist | General practitioner |
| The out-of-pocket costs for this follow-up scenario will be: | 0 euro | 115 euro | 0 euro |
| The duration of the additional follow-up visit(s) will be: | N/A (Self-examination) | 10 minutes | 5 minutes |
| Part of skin to be checked during the additional follow-up visits: | N/A (Self-examination) | Full body | Face, upper body and treated area |
|  | 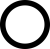 | 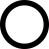 | 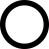 |

| **Question 13** | **Follow-up scenario A** | **Follow-up scenario B** | **Follow-up scenario C** |
| --- | --- | --- | --- |
| Standard post-treatment visit performed: | By the same person as treatment provider | By the same person as treatment provider | Not by same person as treatment provider |
| In addition to oral information, extra information will be provided by: | Personalised letter | E-health | Personalised letter |
| The additional follow-up visit(s) will be planned: | There will be no additional follow-up visit planned. The patient will make an appointment if he or she finds a suspicious lesion. | 6 months and 1 year after treatment | 1 and 2 years after treatment |
| The additional follow-up visit(s) will be conducted by: | N/A  (Self-examination) | General practitioner | Dermatologist |
| The out-of-pocket costs for this follow-up scenario will be: | 0 euro | 0 euro | 230 euro |
| The duration of the additional follow-up visit(s) will be: | N/A (Self-examination) | 10 minutes | 5 minutes |
| Part of skin to be checked during the additional follow-up visits: | N/A (Self-examination) | Full body | Face, upper body and treated area |
|  | 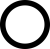 | 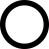 | 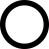 |

| **Question 14** | **Follow-up scenario A** | **Follow-up scenario B** | **Follow-up scenario C** |
| --- | --- | --- | --- |
| Standard post-treatment visit performed: | By the same person as treatment provider | Not by same person as treatment provider | By the same person as treatment provider |
| In addition to oral information, extra information will be provided by: | General hand-out | E-health | Personalised letter |
| The additional follow-up visit(s) will be planned: | There will be no additional follow-up visit planned. The patient will make an appointment if he or she finds a suspicious lesion. | 1 year after treatment | 1 and 2 years after treatment |
| The additional follow-up visit(s) will be conducted by: | N/A  (Self-examination) | Nurse practitioner | General practitioner |
| The out-of-pocket costs for this follow-up scenario will be: | 0 euro | 85 euro | 0 euro |
| The duration of the additional follow-up visit(s) will be: | N/A (Self-examination) | 15 minutes | 10 minutes |
| Part of skin to be checked during the additional follow-up visits: | N/A (Self-examination) | Full body | Face, upper body and treated area |
|  | 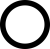 | 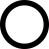 | 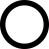 |

| **Question 15** | **Follow-up scenario A** | **Follow-up scenario B** | **Follow-up scenario C** |
| --- | --- | --- | --- |
| Standard post-treatment visit performed: | Not by same person as treatment provider | By the same person as treatment provider | Not by same person as treatment provider |
| In addition to oral information, extra information will be provided by: | General website | General website | E-health |
| The additional follow-up visit(s) will be planned: | There will be no additional follow-up visit planned. The patient will make an appointment if he or she finds a suspicious lesion. | 6 months and 1 year after treatment | 1 and 2 years after treatment |
| The additional follow-up visit(s) will be conducted by: | N/A  (Self-examination) | General practitioner | Dermatologist |
| The out-of-pocket costs for this follow-up scenario will be: | 0 euro | 0 euro | 230 euro |
| The duration of the additional follow-up visit(s) will be: | N/A (Self-examination) | 15 minutes | 10 minutes |
| Part of skin to be checked during the additional follow-up visits: | N/A (Self-examination) | Full body | Face, upper body and treated area |
|  | 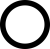 | 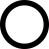 | 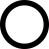 |

| **Question 16** | **Follow-up scenario A** | **Follow-up scenario B** | **Follow-up scenario C** |
| --- | --- | --- | --- |
| Standard post-treatment visit performed: | Not by same person as treatment provider | Not by same person as treatment provider | By the same person as treatment provider |
| In addition to oral information, extra information will be provided by: | Personalised letter | Personalised letter | General hand-out |
| The additional follow-up visit(s) will be planned: | There will be no additional follow-up visit planned. The patient will make an appointment if he or she finds a suspicious lesion. | 1 and 2 years after treatment | 1 year after treatment |
| The additional follow-up visit(s) will be conducted by: | N/A  (Self-examination) | General practitioner | Dermatologist |
| The out-of-pocket costs for this follow-up scenario will be: | 0 euro | 0 euro | 115 euro |
| The duration of the additional follow-up visit(s) will be: | N/A (Self-examination) | 15 minutes | 10 minutes |
| Part of skin to be checked during the additional follow-up visits: | N/A (Self-examination) | Face, upper body and treated area | Full body |
|  | 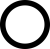 | 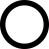 | 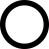 |

| **Question 17** | **Follow-up scenario A** | **Follow-up scenario B** | **Follow-up scenario C** |
| --- | --- | --- | --- |
| Standard post-treatment visit performed: | By the same person as treatment provider | Not by same person as treatment provider | Not by same person as treatment provider |
| In addition to oral information, extra information will be provided by: | General website | Personalised letter | General website |
| The additional follow-up visit(s) will be planned: | There will be no additional follow-up visit planned. The patient will make an appointment if he or she finds a suspicious lesion. | 1 year after treatment | 1 and 2 years after treatment |
| The additional follow-up visit(s) will be conducted by: | N/A  (Self-examination) | Nurse practitioner | Dermatologist |
| The out-of-pocket costs for this follow-up scenario will be: | 0 euro | 85 euro | 230 euro |
| The duration of the additional follow-up visit(s) will be: | N/A (Self-examination) | 5 minutes | 15 minutes |
| Part of skin to be checked during the additional follow-up visits: | N/A (Self-examination) | Full body | Face, upper body and treated area |
|  | 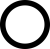 | 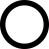 | 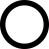 |

| **Question 18** | **Follow-up scenario A** | **Follow-up scenario B** | **Follow-up scenario C** |
| --- | --- | --- | --- |
| Standard post-treatment visit performed: | Not by same person as treatment provider | Not by same person as treatment provider | By the same person as treatment provider |
| In addition to oral information, extra information will be provided by: | General hand-out | E-health | General website |
| The additional follow-up visit(s) will be planned: | There will be no additional follow-up visit planned. The patient will make an appointment if he or she finds a suspicious lesion. | 1 and 2 years after treatment | 1 year after treatment |
| The additional follow-up visit(s) will be conducted by: | N/A  (Self-examination) | Dermatologist | Nurse practitioner |
| The out-of-pocket costs for this follow-up scenario will be: | 0 euro | 230 euro | 85 euro |
| The duration of the additional follow-up visit(s) will be: | N/A (Self-examination) | 5 minutes | 15 minutes |
| Part of skin to be checked during the additional follow-up visits: | N/A (Self-examination) | Full body | Face, upper body and treated area |
|  | 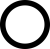 | 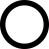 | 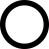 |

| **Question 19** | **Follow-up scenario A** | **Follow-up scenario B** | **Follow-up scenario C** |
| --- | --- | --- | --- |
| Standard post-treatment visit performed: | Not by same person as treatment provider | By the same person as treatment provider | Not by same person as treatment provider |
| In addition to oral information, extra information will be provided by: | Personalised letter | General website | Personalised letter |
| The additional follow-up visit(s) will be planned: | There will be no additional follow-up visit planned. The patient will make an appointment if he or she finds a suspicious lesion. | 6 months and 1 year after treatment | 1 and 2 years after treatment |
| The additional follow-up visit(s) will be conducted by: | N/A  (Self-examination) | Dermatologist | Nurse practitioner |
| The out-of-pocket costs for this follow-up scenario will be: | 0 euro | 115 euro | 170 euro |
| The duration of the additional follow-up visit(s) will be: | N/A (Self-examination) | 5 minutes | 15 minutes |
| Part of skin to be checked during the additional follow-up visits: | N/A (Self-examination) | Face, upper body and treated area | Full body |
|  | 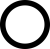 | 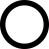 | 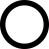 |

| **Question 20** | **Follow-up scenario A** | **Follow-up scenario B** | **Follow-up scenario C** |
| --- | --- | --- | --- |
| Standard post-treatment visit performed: | By the same person as treatment provider | By the same person as treatment provider | Not by same person as treatment provider |
| In addition to oral information, extra information will be provided by: | General website | E-health | Personalised letter |
| The additional follow-up visit(s) will be planned: | There will be no additional follow-up visit planned. The patient will make an appointment if he or she finds a suspicious lesion. | 1 and 2 years after treatment | 6 months and 1 year after treatment |
| The additional follow-up visit(s) will be conducted by: | N/A  (Self-examination) | Nurse practitioner | General practitioner |
| The out-of-pocket costs for this follow-up scenario will be: | 0 euro | 170 euro | 0 euro |
| The duration of the additional follow-up visit(s) will be: | N/A (Self-examination) | 10 minutes | 5 minutes |
| Part of skin to be checked during the additional follow-up visits: | N/A (Self-examination) | Face, upper body and treated area | Full body |
|  | 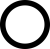 | 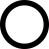 | 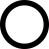 |

| **Question 21** | **Follow-up scenario A** | **Follow-up scenario B** | **Follow-up scenario C** |
| --- | --- | --- | --- |
| Standard post-treatment visit performed: | Not by same person as treatment provider | By the same person as treatment provider | Not by same person as treatment provider |
| In addition to oral information, extra information will be provided by: | E-health | General website | General hand-out |
| The additional follow-up visit(s) will be planned: | There will be no additional follow-up visit planned. The patient will make an appointment if he or she finds a suspicious lesion. | 1 and 2 years after treatment | 6 months and 1 year after treatment |
| The additional follow-up visit(s) will be conducted by: | N/A  (Self-examination) | Nurse practitioner | General practitioner |
| The out-of-pocket costs for this follow-up scenario will be: | 0 euro | 170 euro | 0 euro |
| The duration of the additional follow-up visit(s) will be: | N/A (Self-examination) | 5 minutes | 10 minutes |
| Part of skin to be checked during the additional follow-up visits: | N/A (Self-examination) | Full body | Face, upper body and treated area |
|  | 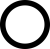 | 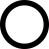 | 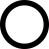 |

| **Question 22** | **Follow-up scenario A** | **Follow-up scenario B** | **Follow-up scenario C** |
| --- | --- | --- | --- |
| Standard post-treatment visit performed: | Not by same person as treatment provider | Not by same person as treatment provider | By the same person as treatment provider |
| In addition to oral information, extra information will be provided by: | E-health | General website | Personalised letter |
| The additional follow-up visit(s) will be planned: | There will be no additional follow-up visit planned. The patient will make an appointment if he or she finds a suspicious lesion. | 6 months and 1 year after treatment | 1 year after treatment |
| The additional follow-up visit(s) will be conducted by: | N/A  (Self-examination) | General practitioner | Nurse practitioner |
| The out-of-pocket costs for this follow-up scenario will be: | 0 euro | 0 euro | 85 euro |
| The duration of the additional follow-up visit(s) will be: | N/A (Self-examination) | 5 minutes | 15 minutes |
| Part of skin to be checked during the additional follow-up visits: | N/A (Self-examination) | Face, upper body and treated area | Full body |
|  | 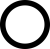 | 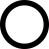 | 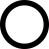 |

| **Question 23** | **Follow-up scenario A** | **Follow-up scenario B** | **Follow-up scenario C** |
| --- | --- | --- | --- |
| Standard post-treatment visit performed: | By the same person as treatment provider | By the same person as treatment provider | Not by same person as treatment provider |
| In addition to oral information, extra information will be provided by: | General hand-out | General hand-out | General website |
| The additional follow-up visit(s) will be planned: | There will be no additional follow-up visit planned. The patient will make an appointment if he or she finds a suspicious lesion. | 1 year after treatment | 6 months and 1 year after treatment |
| The additional follow-up visit(s) will be conducted by: | N/A  (Self-examination) | Nurse practitioner | Dermatologist |
| The out-of-pocket costs for this follow-up scenario will be: | 0 euro | 85 euro | 115 euro |
| The duration of the additional follow-up visit(s) will be: | N/A (Self-examination) | 15 minutes | 5 minutes |
| Part of skin to be checked during the additional follow-up visits: | N/A (Self-examination) | Face, upper body and treated area | Full body |
|  | 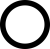 | 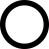 | 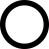 |

| **Question 24** | **Follow-up scenario A** | **Follow-up scenario B** | **Follow-up scenario C** |
| --- | --- | --- | --- |
| Standard post-treatment visit performed: | By the same person as treatment provider | Not by same person as treatment provider | By the same person as treatment provider |
| In addition to oral information, extra information will be provided by: | E-health | General hand-out | E-health |
| The additional follow-up visit(s) will be planned: | There will be no additional follow-up visit planned. The patient will make an appointment if he or she finds a suspicious lesion. | 6 months and 1 year after treatment | 1 year after treatment |
| The additional follow-up visit(s) will be conducted by: | N/A  (Self-examination) | Nurse practitioner | General practitioner |
| The out-of-pocket costs for this follow-up scenario will be: | 0 euro | 85 euro | 0 euro |
| The duration of the additional follow-up visit(s) will be: | N/A (Self-examination) | 10 minutes | 5 minutes |
| Part of skin to be checked during the additional follow-up visits: | N/A (Self-examination) | Face, upper body and treated area | Full body |
|  | 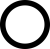 | 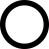 | 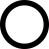 |

| **Question 25** | **Follow-up scenario A** | **Follow-up scenario B** | **Follow-up scenario C** |
| --- | --- | --- | --- |
| Standard post-treatment visit performed: | By the same person as treatment provider | Not by same person as treatment provider | By the same person as treatment provider |
| In addition to oral information, extra information will be provided by: | General website | General hand-out | General website |
| The additional follow-up visit(s) will be planned: | There will be no additional follow-up visit planned. The patient will make an appointment if he or she finds a suspicious lesion. | 1 year after treatment | 6 months and 1 year after treatment |
| The additional follow-up visit(s) will be conducted by: | N/A  (Self-examination) | Dermatologist | Nurse practitioner |
| The out-of-pocket costs for this follow-up scenario will be: | 0 euro | 115 euro | 85 euro |
| The duration of the additional follow-up visit(s) will be: | N/A (Self-examination) | 15 minutes | 10 minutes |
| Part of skin to be checked during the additional follow-up visits: | N/A (Self-examination) | Full body | Face, upper body and treated area |
|  | 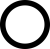 | 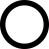 | 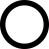 |
